# Untangling the Association Between Age, Race, Family help and Self-reported Stress Among Dementia Caregivers

**DOI:** 10.1093/geroni/igaf122.2929

**Published:** 2025-12-31

**Authors:** Obinna Odo, Tochukwu Ozor

**Affiliations:** Miami University, Oxford, Ohio, United States; Nnamdi Azikiwe University, Nnewi, Anambra, Nigeria

## Abstract

Studies show that providing care to older adults with Alzheimer’s Disease and Other Related Dementias [ADRD] is stressful because the care needs are often challenging and extensive. Self-reported caregiving stress is someone’s cognitive appraisal of threats emanating from caregiving stressors such that the demands of the stressors outweigh the individual’s available resources. Caregiving stress results in loss of leisure time, emotional exhaustion, and work overload, affecting caregivers’ physical and emotional health. This study examined factors that affect self-reported stress among caregivers of older adults with ADRD. Data from the 2017 National Study on Caregiving (NSOC III round 7) and the NSOC Time data subset were merged (N = 1341; Mean age = 58.3) and analyzed using bivariate and multiple linear regression analysis. Among caregivers who identified as Black, dementia caregiving was associated with a decrease in self-reported stress (β = -0.095, p < 0.05). Age was also negatively associated with self-reported stress, with higher age predicting lower caregiving stress (β = -0.007, p < .001). Feelings of frustration was strongly associated with caregiving stress (β = 0.492, p < .001). Surprisingly, access to family help was associated with higher caregiving stress (β = 0.12, p < 0.05). These findings underscore the need for tailored caregiving interventions and programs that address the unique needs of dementia caregivers, such as respite care and mental health support services, to mitigate emotional exhaustion, anxiety, and burden. The study also highlights the need for family caregiver training aimed at easing the stress on dementia primary caregivers.

